# Nuclear 2′-*O*-methylation regulates RNA splicing through its binding protein FUBP1

**DOI:** 10.1126/sciadv.ady3894

**Published:** 2025-10-17

**Authors:** Boyang Gao, Bochen Jiang, Zhongyu Zou, Bei Liu, Weijin Liu, Li Chen, Lisheng Zhang, Chuan He

**Affiliations:** ^1^ Department of Molecular Genetics and Cell Biology, University of Chicago, Chicago, IL 60637, USA.; ^2^ Howard Hughes Medical Institute, The University of Chicago, Chicago, IL 60637, USA.; ^3^ Department of Chemistry, The University of Chicago, Chicago, IL 60637, USA.; ^4^ Division of Life Science, The Hong Kong University of Science and Technology (HKUST), Kowloon, Hong Kong SAR, China.; ^5^ Department of Chemistry, The Hong Kong University of Science and Technology (HKUST), Kowloon, Hong Kong SAR, China.

## Abstract

2′-*O*-methylation (N_m_) is an abundant RNA modification exists on different mammalian RNA species. However, potential N_m_ recognition by proteins has not been extensively explored. Here, we used RNA affinity purification, followed by mass spectrometry to identify N_m_-binding proteins. The N_m_-binding protein candidates exhibit enriched binding at known N_m_ sites. Some candidates display nuclear localization and functions. We focused on the splicing factor FUBP1. Electrophoretic mobility shift assay validated preference of FUBP1 to N_m_-modified RNA. As FUBP1 predominantly binds intronic regions, we profiled N_m_ sites in chromatin-associated RNA (caRNA) and found N_m_ enrichment within introns. Depletion of N_m_ led to skipped exons, suggesting N_m_-dependent splicing regulation. The caRNA N_m_ sites overlap with FUBP1-binding sites, and N_m_ depletion reduced FUBP1 occupancy on modified regions. Furthermore, *FUBP1* depletion induced exon skipping in N_m_-modified genes, supporting its role in mediating N_m_-dependent splicing regulation. Overall, our findings identify FUBP1 as an N_m_-binding protein and uncover previously unrecognized nuclear functions for RNA N_m_ modification.

## Teaser

FUBP1 is identified as an N_m_-binding protein that regulates N_m_-dependent splicing via intronic sites on chromatin-associated RNA.

## INTRODUCTION

Throughout the life cycles of RNA, chemical modifications play critical roles in regulating RNA processing, metabolism, and function (*1*, *2*). These modifications can alter the physical properties of the RNA molecule (*3*, *4*) or recruit specific binding proteins (readers) to modulate RNA function and subsequent cellular pathways (*5*–*9*). While changes in physical properties primarily influence RNA structure–dependent regulation, the recruitment of reader proteins can affect diverse downstream processes, including splicing (*10*), degradation (*5*), translation (*6*, *9*), and RNA transport to specialized cellular locations (*11*–*13*). In addition, recruitment of binding proteins may alter the surrounding state of the modified RNA; examples include chromatin state regulation by *N*^6^-methyladenosine (m^6^A) methylation of chromatin-associated RNA (caRNA) (*7*, *8*, *14*).

2′-*O*-methylation (N_m_) is one of the most abundant modifications (*15*). It can be found in almost all RNA species, including ribosomal RNA (rRNA), tRNA, small nuclear RNA, small nucleolar RNA (snoRNA), microRNA, and mRNA. It has been shown to regulate ribosome biogenesis (*16*, *17*), gene expression (*18*, *19*), innate immune sensing (*20*, *21*), and cell fate decisions (*22*). N_m_ entails methylation at the 2′-OH position of the ribose on the RNA backbone. Consequently, it can occur on any of the four ribonucleotide residues, namely, 2′-*O*-methyladenosine (A_m_), 2′-*O*-methylguanosine (G_m_), 2′-*O*-methylcytidine (C_m_), and 2′-*O*-methyluridine (U_m_). The methylation further stabilizes the ribose 3′-endo conformation, favoring the A-type RNA helix and restricting strand flexibility (*23*–*25*). Consequently, N_m_ installation alters physical properties of the modified RNA. This effect orchestrates N_m_-dependent cellular functions of different RNA species, such as stabilizing ribosome structure through modified rRNA (*26*) and controlling translational efficiency through internal mRNA N_m_ modification (*18*). However, whether N_m_ could be recognized by binding proteins (readers) remains largely unexplored, particularly within the internal regions of mRNA or premature mRNA. While previous studies have identified over a thousand internal N_m_ sites (*27*), the corresponding function remains elusive. The processing, metabolism, or function of N_m_-modified RNA could be unveiled by the identification of N_m_-binding proteins. A previous study reported a functional relationship between cleavage and polyadenylation specificity factor subunit 7 (CPSF7) binding and N_m_-mediated alternative polyadenylation, suggesting CPSF7 as a potential N_m_-binding protein (*28*). However, more comprehensive identification of N_m_-binding proteins is still lacking.

In this work, we conducted RNA affinity purification, followed by mass spectrometry (MS) to identify N_m_-binding protein candidates, among which we focused on the splicing factor, far upstream element binding protein 1 (FUBP1). Up-regulation of *FUBP1* has been suggested to promote proliferation of multiple types of cancers (*29*, *30*). Initially characterized as a transcription factor modulating MYC expression, FUBP1 is now recognized as an RNA binding protein (RBP) involved in pre-mRNA splicing (*31*, *32*). Previous studies have indicated that FUBP1 stabilizes splicing machineries at 3′ splice sites, including U2AF2 and SF1. In addition, it interacts with components of U1 small nuclear ribonucleoproteins, potentially facilitating splice sites pairing in long introns. Understanding the binding specificity of FUBP1 could elucidate the mechanism underlying alternative splicing (AS) events that could be affected by N_m_ modification through FUBP1.

Using electrophoretic mobility shift assay (EMSA), we confirmed the binding preference of FUBP1 to N_m_-modified oligos, supporting its role as an N_m_-binding protein. N_m_-mut-seq (*27*) analysis of caRNA in HepG2 cells identified 5575 N_m_ sites, with more than half of the intragenic N_m_ sites localized in introns. Disruption of N_m_ installation led to altered splicing patterns, especially increased exon skipping. These N_m_ sites were bound by FUBP1 in a manner responsive to N_m_ depletion. Last, FUBP1 preferentially regulates exon skipping at N_m_-modified regions. Together, our study identifies FUBP1 as one of the first examples of N_m_-binding proteins and highlights a previously unrecognized role of N_m_ in splicing regulation.

## RESULTS

### RNA affinity purification followed by MS identified N_m_-binding protein candidates

**Fig. 1.**
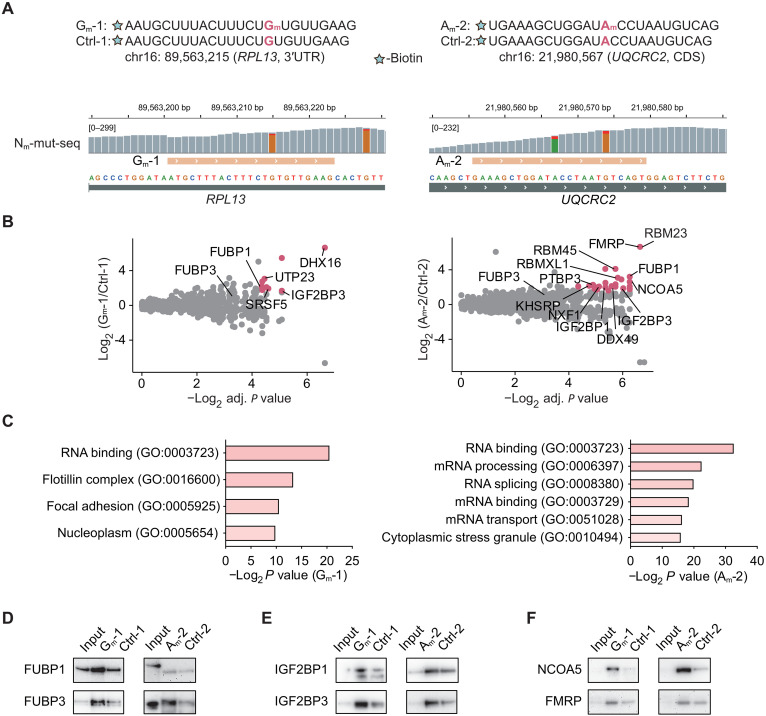
RNA affinity purification followed by proteomics identified N_m_-binding protein candidates. (**A**) Design of G_m_-1 and A_m_-2 probes based on known N_m_ sites in HepG2 mRNA. Integrative genomics viewer (IGV) tracks of published N_m_-mut-seq showed read depth of mutated (red) and unmutated reads (G in orange and A in green). (**B**) Proteins that may preferentially bind to RNA G_m_-1 (left) and A_m_-2 (right) probes over control unmodified probes. Tentative N_m_-binding proteins (red) have a log_2_ fold change of >1.58, an adjusted *P* < 0.05, and a peptide number of ≥4. (**C**) Gene ontology (GO) analysis of tentative N_m_-binding proteins. (**D** to **F**) Immunoblotting of N_m_-binding protein candidate enrichment by oligo pull-down. The ratio of the corresponding lysate amount of input versus pull-down samples are 1:100.

To identify proteins that may preferentially recognize internal RNA N_m_ modification, we designed biotinylated RNA probes based on published internal N_m_ sites in HepG2 (*27*). Given that N_m_ can occur on four distinct types of ribonucleotides (A_m_, G_m_, C_m_, and U_m_), probes with a single type of N_m_ may not fully capture the binding proteins recognizing four different N_m_ modifications. Considering the predominant presence of G_m_ and A_m_ in mRNA N_m_ modifications (*27*), we designed two probes: one with G_m_ modification (G_m_-1) and the other with A_m_ modification (A_m_-2) (Fig. 1A). Each probe was accompanied by a corresponding control oligo lacking the N_m_ modification, denoted as Ctrl-1 and Ctrl-2. These probes were selected on the basis of two reported N_m_ sites, with relatively high mutation ratio and responsiveness to the knockdown (KD) of their methyltransferase fibrillarin (FBL; Fig. 1) (*27*). The G_m_-1 sequence is in the 3′ untranslated region (3′UTR) of *RPL13*, while the A_m_-2 sequence situates in the coding sequence (CDS) of *UQCRC2*.

We performed affinity purification with G_m_-1 and A_m_-2 using HepG2 cell lysate, respectively, and used MS for protein identification of the pull-down fraction. Excess probes were used in these experiments (fig. S1A). Peptide abundance was normalized to the total peptide abundance, and fold changes of N_m_-modified versus control groups were computed. With a cutoff of a log_2_ fold change of >1.58, an adjusted *P* < 0.05, and a peptide number ≥ 4, we identified 37 enriched proteins in the G_m_-1 group and 33 enriched proteins in A_m_-2 group (Fig. 1B and fig. S1B). These enriched proteins are tentative binding proteins for G_m_ and A_m_ modifications. The identified protein candidates include mRNA binding proteins and proteins involved in RNA-related metabolic pathways, based on gene ontology (GO) analysis (Fig. 1C), validating the reliability of our assay.

In the candidate protein list, we observed proteins present in both G_m_- and A_m_-enriched groups, indicating them as potential N_m_ binders without base specificity. One example is FUBP1; another homolog within the same protein family, FUBP3, is also weakly enriched by both oligo pairs. The structural similarity within the protein family suggests that both two proteins may preferentially bind to N_m_. To validate this speculation, we detected the protein enrichment by immunoblotting after RNA affinity purification using the two pairs of probes (Fig. 1A). As expected, both FUBP1 and FUBP3 were enriched by the two N_m_ probes compared to their respective controls (Fig. 1D). This observation indicates that FUBP1 and FUBP3 are tentative candidates recognizing both G_m_ and A_m_ modifications on RNA.

In addition, insulin-like growth factor 2 mRNA binding protein 1 (IGF2BP1) and IGF2BP3 emerged as candidate binders of G_m_ and A_m_. IGF2BPs were initially identified as m^6^A-binding proteins that stabilize target mRNAs and facilitates their storage under stress conditions (*33*). Our previous work also revealed their role in recognizing 7-methylguanosine (m^7^G) (*34*). While IGF2BP1 primarily binds m^6^A and stabilizes associated mRNAs, IGF2BP3 preferentially recognizes m^7^G and promotes mRNA degradation. Immunoblotting following affinity purification of both probe pairs confirmed the preferentially binding of IGF2BP1 and IGF2BP3 to N_m_-modified RNA (Fig. 1E). These findings may suggest a complex mechanism of RNA modification recognition by the IGF2BP proteins.

In addition to the FUBP and IGF2BP families, we also validated enrichment of fragile X messenger ribonucleoprotein 1 (FMRP) and nuclear receptor coactivator 5 (NCOA5). FMRP and NCOA5 were enriched by both G_m_-1/Ctrl-1 and A_m_-2/Ctrl-2 probe pairs through affinity purification, followed by immunoblotting (Fig. 1F). The RBP FMRP has previously been shown to colocalize with internal N_m_ sites (*28*). Our validation by RNA affinity purification further supports its tentative role as an N_m_-binding protein.

Notably, NCOA5 is a transcriptional coactivator that interacts with estrogen receptors (*35*). The enrichment of NCOA5 by RNA affinity purification suggests it as another example of a transcription factor that binds to and is potentially regulated by RNA. We analyzed a published RNA binding region identification dataset (*36*) and observed enriched NCOA5 peptides, indicating its involvement in RNA binding as an RBP (fig. S1C). Its preferential binding to N_m_ may suggest a possible role for N_m_ in transcriptional regulation. Overall, we validated six proteins with enriched binding to N_m_-modified probes: FUBP1, FUBP3, IGF2BP1, IGF2BP3, FMRP, and NCOA5.

### N_m_-binding protein candidates are enriched at internal mRNA N_m_ sites

**Fig. 2.**
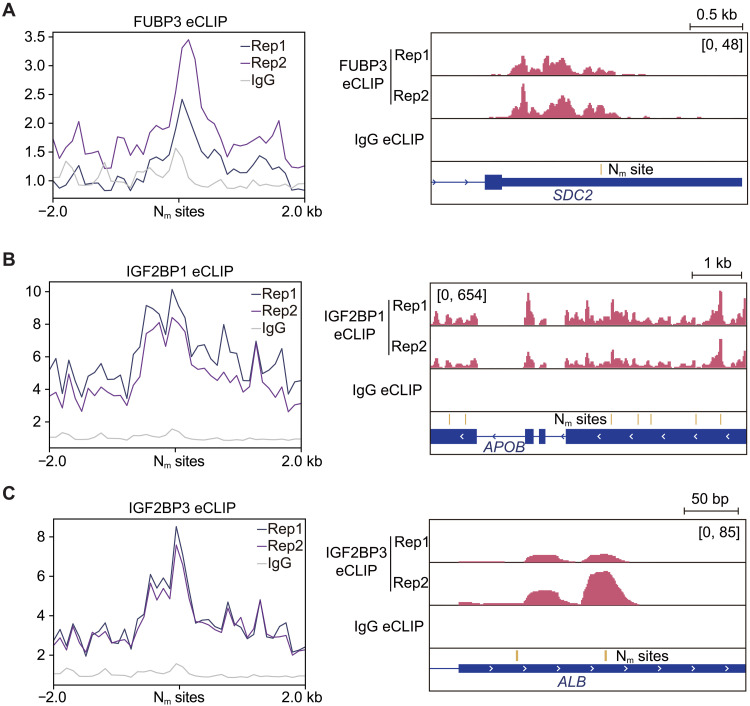
RNA binding sites of N_m_-binding protein candidates were enriched at N_m_ sites. (**A** to **C**) Metagene plots of eCLIP signals at HepG2 mRNA N_m_ sites (left) and representative IGV tracks (right) from binding sites of N_m_ binding protein candidates FUBP3 (A), IGF2BP1 (B), and IGF2BP3 (C). Ranges of the signal intensity are annotated in the brackets.

To provide further cellular evidence of the association between binding of these proteins and N_m_ modifications, we investigated their RNA binding profiles using publicly available eCLIP datasets from the ENCODE project (*37*, *38*). Among the six identified tentative N_m_ binders, FUBP3, IGF2BP1, and IGF2BP3 have eCLIP data generated in HepG2 cells. Metagene plots generated from these datasets revealed enriched binding of all four proteins around the reported confident N_m_ sites (Fig. 2, A to C) (*27*), supporting the association of binding by these proteins with N_m_ modification. This is consistent with previous reports, where IGF2BP1 and IGF2BP3 have also been suggested for their colocalization with internal N_m_ sites in other cell lines (*28*). In vitro validation of the direct binding preference is still lacking for these three proteins, so their roles as N_m_-binding proteins require further validations.

We examined the sequence contexts of the three proteins at their N_m_-associated binding sites. The enriched N_m_ motifs from the overlapped binding sites closely resembled their own RNA binding motifs (fig. S2, A to C), suggesting that the binding selectivity of these N_m_-binding protein candidates are determined by their canonical sequence contexts but may be further enhanced by the N_m_ modification. To further investigate how RNA binding preferences affect potential N_m_ site selectivity, we analyzed the distribution of their binding targets and associated N_m_ sites across mRNA regions. While N_m_ sites were generally enriched in the CDS compared to the 3′UTR and 5′UTR, N_m_-binding protein with 3′UTR preferences, FUBP3, predominantly bound to N_m_ sites within the 3′UTR, highlighting its preferences to its canonical binding sites (fig. S2D). Conversely, IGF2BP1 and IGF2BP3 exhibited a stronger enrichment of bound N_m_ sites in the CDS than their overall binding sites (fig. S2D), suggesting a potentially important contribution of selective N_m_ installation to RNA binding by these RBPs.

The reported function of IGF2BP1 in stabilizing mRNA aligns with the general effect of N_m_ on mRNA levels as previously described (*19*, *27*, *28*). Accordingly, we analyzed RNA level changes using published KD datasets for *IGF2BP1* in HepG2 cells (*33*). N_m_-modified mRNA transcripts showed a more notable decrease in expression following *IGF2BP1* KD compared to unmodified transcripts (fig. S2E), supporting the association between IGF2BP1 binding and N_m_ modifications. Further investigation into IGF2BP1 binding to methylated RNA is needed to elucidate the interplay among m^6^A, m^7^G, and N_m_ modifications.

### FUBP1 preferentially binds internal mRNA N_m_ sites

**Fig. 3.**
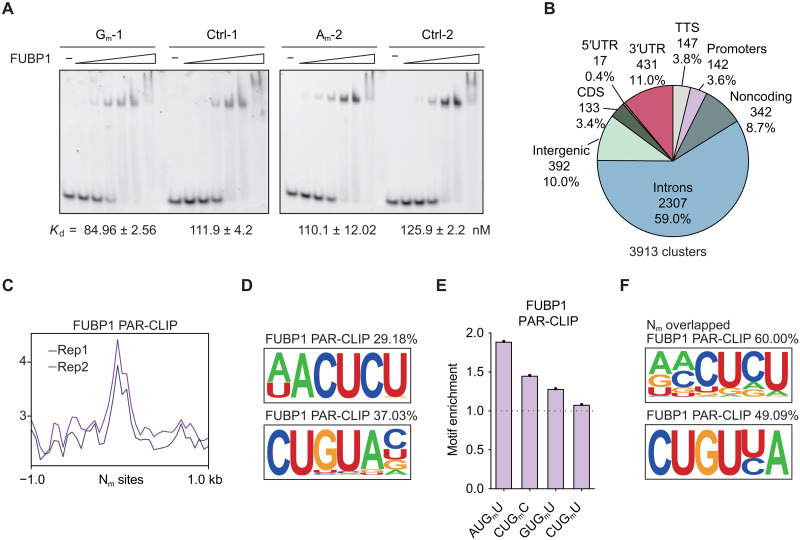
Splicing regulator FUBP1 prefers N_m_-modified RNA. (**A**) EMSA of FUBP1 binding toward G_m_-1 and A_m_-2 probes versus their controls. Probe concentration, 20 nM; protein concentration, starting from 400 nM with twofold dilution. (**B**) Distribution of FUBP1 PAR-CLIP binding clusters across transcript elements. TTS, transcription termination sites. (**C**) Metagene plot of FUBP1 PAR-CLIP signals at HepG2 mRNA N_m_ sites. (**D**) FUBP1-binding motifs identified in PAR-CLIP. (**E**) Enrichment of N_m_ motifs at the FUBP1-binding sites. (**F**) FUBP1-binding motifs identified in PAR-CLIP clusters overlapping with mRNA N_m_ sites.

The enrichment of nuclear proteins in the N_m_ binder candidate list suggests intriguing functions of N_m_ in the cell nucleus. We chose FUBP1 as an example for more detailed investigations, as FUBP1 is a well-characterized splicing factor (*29*, *32*). Its preference for N_m_ has been validated using the two probe pairs in both proteomics and immunoblotting analyses. Nevertheless, the RNA affinity purification approach leaves a possibility of indirect FUBP1 binding mediated by an N_m_-recognizing partner protein. To confirm the direct interaction between FUBP1 and the N_m_ modification, we recombinantly expressed and purified FUBP1 with a C-terminal strep-tag from the Expi293 expression system (fig. S3A). We then assessed the binding affinity of the purified FUBP1 toward two probe pairs by EMSA. We observed preferential binding of FUBP1 to both N_m_-modified RNA probes compared to their respective controls (Fig. 3A). As a sugar 2′-OH modification, N_m_ recognition by FUBP1 could be more subtle, when compared with other well-recognized RNA modifications such as m^6^A by the reader YTH N6-methyladenosine RNA binding proteins (YTHDF proteins) (*5*). Our EMSA results support that the preferential binding of FUBP1 to N_m_ is mediated by a direct interaction. Future structural characterization may reveal details of this recognition.

In addition to biochemical evidence, we examined whether FUBP1 binding occurs at endogenous N_m_ sites within various RNA sequence contexts. We conducted photoactivatable ribonucleoside-enhanced cross-linking and immunoprecipitation (PAR-CLIP) of FUBP1 in HepG2 cells. Genome-wide mutation analysis revealed a T-to-C mutation ratio of >55% (fig. S3B). We identified 3913 FUBP1-binding sites (fig. S3C), with ~59% of these located within intronic regions (Fig. 3B), consistent with the reported intron preference of FUBP1 binding (*32*). With the published mRNA N_m_ sites at base resolution, we first investigated FUBP1 binding at reported mature mRNA N_m_ sites. A metagene plot revealed a clear enrichment of PAR-CLIP reads at the confident N_m_ sites (Fig. 3C). FUBP1-binding motifs were found to be U-rich (Fig. 3D), consistent with the U-rich motifs associated with mRNA N_m_. Notably, N_m_ motifs enriched at FUBP1-binding sites closely resembled the RNA binding motifs shared across all its binding sites (Fig. 3, E and F). These results indicate an intrinsic preference of FUBP1 for N_m_-modified regions, further supporting its role as an N_m_-binding protein.

### Abundant N_m_ modifications on caRNA regulate splicing events

**Fig. 4.**
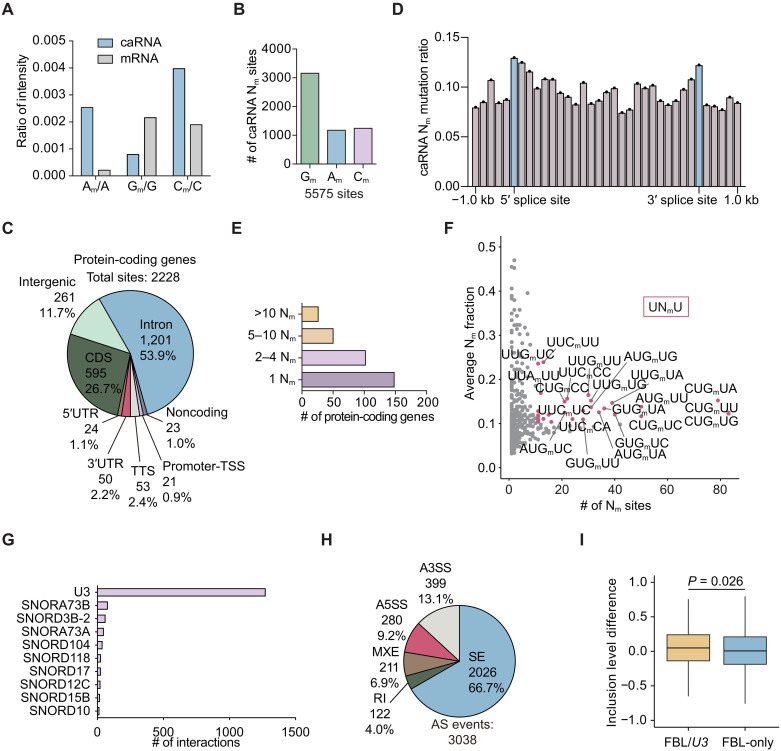
N_m_ modifications on HepG2 caRNA affect splicing events. (**A**) UHPLC-QQQ-MS/MS of N_m_ abundances in HepG2 mRNA and caRNA. (**B**) Composition of G_m_, A_m_, and C_m_ among the 5575 caRNA N_m_ sites. (**C**) Transcript element distribution of 2228 caRNA N_m_ sites in protein-coding genes. (**D**) Averaged caRNA N_m_ mutation ratio across intronic regions in a 200-bp binning. Intronic sequences at the boundary of splice sites were marked in blue. (**E**) Profile of modified protein-coding gene transcripts with the corresponding numbers of N_m_ sites. (**F**) Number and average mutation ratio of all five-base N_m_ motifs. UN_m_U was enriched in more frequent and highly modified motifs. (**G**) Number of interactions with various snoRNA detected by snoKARR-seq at all caRNA N_m_ sites. (**H**) Profile of FBL-dependent AS events, with a significance threshold false discovery rate (FDR) of <0.1. (**I**) Inclusion level differences of FBL/*U3* or FBL-only SE events.

The preference of splice factor FUBP1 for N_m_ modifications indicates an unrecognized intron-dependent function of N_m_. To explore whether N_m_ modifications are also present in intronic regions, we measured the overall levels of N_m_ in rRNA-depleted HepG2 caRNA by ultrahigh-performance liquid chromatography coupled with triple quadrupole MS (UHPLC-QQQ-MS/MS), with quantitative polymerase chain reaction (qPCR) validation of the rRNA contamination comparable to or lower than mRNA (fig. S4A). The intensity ratios of A_m_/A and C_m_/C are notably higher in caRNA compared to mRNA (Fig. 4A). The G_m_/G ratio in caRNA is about one-third of that in mRNA; however, the considerably longer length of introns likely compensates, resulting in an overall more G_m_ sites in introns than those in exons. Our findings suggest enrichment of N_m_ modifications in caRNA.

To examine caRNA N_m_ distribution profiles, we performed N_m_-mut-seq on HepG2 caRNA. N_m_-mut-seq uses an imbalanced deoxynucleotide triphosphate (dNTP) supply in reverse transcription (RT) reactions to induce mutations of G_m_, A_m_, and C_m_ into T (*27*). During analysis, the treated samples could be controlled by RT reactions using balanced dNTP supply (input), as well as a set of spike-in that does not harbor N_m_ modifications but undergoes RT with imbalanced dNTP (background). We analyzed the mutations of caRNA N_m_-mut-seq by JACUSA (*39*) and designated sites as N_m_-modified when all three replicates showed a sequencing depth of ≥10, a mutated read depth of ≥3, mutation ratios of >3-fold of input mutation ratios, and >1.5-fold of background mutation ratios. On the basis of these criteria, we identified 5575 N_m_ sites (fig. S4B), far more than the reported 1051 N_m_ sites or 494 confident N_m_ sites found in HepG2 mRNA (*27*).

Similar to mRNA, G_m_ sites are the most abundantly modified N_m_ sites in caRNA (Fig. 4B), despite the relatively higher abundances of A_m_ and C_m_ detected by UHPLC-QQQ-MS/MS (Fig. 4A). The numbers of confident A_m_ and C_m_ sites in caRNA are comparable. Most of caRNA N_m_ sites are located within protein-coding genes (fig. S4C), with ~53.9% of them residing in introns (Fig. 4C). This is consistent with our hypothesis of intron-dependent function of N_m_. These intronic N_m_ sites show modest enrichment near 5′ and 3′ splice sites (Fig. 4D), suggesting a potential role of N_m_ in splicing regulation. Our previous work has shown that internal N_m_ sites on mRNA often appear densely clustered along a stretch of RNA (*27*). We hypothesized that this clustering may amplify the relatively weak binding preferences of N_m_-binding proteins toward modified RNA. We observed that 2228 caRNA N_m_ sites on protein-coding genes were distributed across only 323 genes, with more than half of these genes harboring multiple N_m_ sites (Fig. 4E). In addition to protein-coding genes, several long noncoding RNAs (lncRNAs) also accumulate hundreds of N_m_ sites (fig. S4D), suggesting a previously unrecognized layer of N_m_-dependent regulation in their respective functions.

N_m_ installation in HepG2 caRNA is enriched in the UN_m_U sequence context (Fig. 4F), similar to the motifs observed in HepG2 mRNA (*27*). The consistency should be expected, as caRNA modifications in intronic regions and noncoding RNA (ncRNA) are generally installed by the same methyltransferases responsible for mRNA modification, despite their different fates after RNA processing and nuclear export (*7*). The observed motifs also overlap with U-rich elements recognized by key splicing factors, such as the 5′ splice sites GU, the branch sites, the UGCAUG hexanucleotides (*40*), the polypyrimidine tract (*41*), etc. This further indicates a role of N_m_ in splicing regulation. Notably, the U-rich motif is consistent with the known RNA binding preference of FUBP1 (*32*), further suggesting its role as an N_m_-binding protein that recognizes intronic N_m_ to potentially modulate splicing.

To understand the regulation of N_m_ installation on caRNA, we analyzed snoRNA interactions with N_m_-modified regions using the published snoRNA enriched and kethoxal assisted RNA-RNA interaction sequencing (snoKARR-seq) dataset (*42*). Most of snoRNA interaction with caRNA N_m_ sites were mediated through *U3* (Fig. 4G and fig. S4E), highlighting the critical role of *U3* in directing N_m_ installation on caRNA. Our finding suggests that *U3* KD may serve as an effective strategy to perturb caRNA N_m_ installation in addition to depletion of the N_m_ methyltransferase *FBL*.

The preference of N_m_ binding by the splicing factor FUBP1 may suggest the potential role of N_m_ in splicing regulation. It has been reported that ectopic overexpression of snoRNA that installs N_m_ in pre-mRNA branch sites could manipulate splicing of certain RNA transcripts (*43*, *44*). To investigate the role of caRNA N_m_ in splicing regulation, we depleted *FBL* or *U3* in HepG2 cells (fig. S4F). We examined five types of AS events: alternative 3′ splice sites (A3SSs), alternative 5′ splice sites (A5SSs), mutually exclusive exons (MXEs), retained introns (RIs), and skipped exons (SEs). AS events dependent on FBL or *U3* exhibited similar distributions, with SE events being the most prevalent (Fig. 4H and fig. S4G). In both *FBL* depletion and *U3* KD, more SE events were up-regulated than down-regulated, while other AS types showed nonsignificant or inconsistent changes (fig. S4H). This indicates that N_m_ installation may play a role in preventing exon skipping. To further examine this, we compared SE events that consistently occurred following both *FBL* and *U3* KD (FBL/*U3*) with those that occurred only or inconsistently after *FBL* KD (FBL-only) or *U3* KD (U3-only). The FBL/*U3* SE events exhibited a higher degree of up-regulation compared to FBL-only and *U3*-only groups (Fig. 4I and fig. S4I), further supporting a role for N_m_ in suppressing SE events. Overall, our findings reveal abundant N_m_ modifications in caRNA, with intronic installation affecting splicing regulation.

### FUBP1 mediates N_m_-dependent splicing regulation

**Fig. 5.**
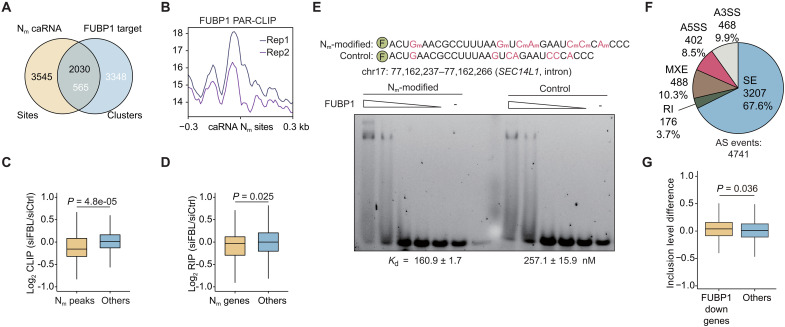
FUBP1 binds to N_m_-modified caRNA to affect splicing. (**A**) Overlap between caRNA N_m_ sites and FUBP1 PAR-CLIP clusters extending 200 bp on each side. (**B**) Metagene plot of FUBP1 PAR-CLIP signals of ±0.3 kb around HepG2 caRNA N_m_ sites. (**C**) FUBP1 CLIP signal changes at peaks overlapping with N_m_ sites (N_m_ peaks) versus others after *FBL* KD. (**D**) FUBP1 RIP signal changes at gene transcripts with N_m_ modifications (N_m_ genes) versus others after *FBL* KD. (**E**) EMSA of FUBP1 binding toward N_m_-modified or control probes originated from *SEC14L1* intron. Probe concentration, 10 nM; protein concentration, starting from 400 nM with twofold dilution. (**F**) Profile of the FUBP1-dependent AS events. (**G**) Inclusion level differences of SE events after FUBP1 depletion in genes with down-regulated FUBP1 CLIP-seq peaks after si*FBL* (FUBP1 down genes) versus other genes (others).

Our N_m_-mut-seq analysis of HepG2 caRNA revealed a substantial presence of N_m_ modifications within intronic regions, potentially involved in splicing regulation. Given that FUBP1 is a known splicing factor implicated in splice site recognition (*32*), we hypothesized that it may mediate N_m_-dependent splicing regulation. To investigate this, we first validated the association of FUBP1 with caRNA N_m_ sites. Of the 5575 caRNA N_m_ sites, 2030 were bound by FUBP1, as determined by PAR-CLIP analysis (Fig. 5A). Correspondingly, FUBP1 PAR-CLIP signals were enriched around caRNA N_m_ sites (Fig. 5B). Although FUBP1 primarily targets protein-coding genes (fig. S3C), it also binds to the heavily modified lncRNAs *MALAT1* and *NEAT1* in multiple clusters (fig. S5A). Together, these findings demonstrate that FUBP1 preferentially binds to N_m_ sites in both caRNA and mRNA, consolidating its role as an N_m_-binding protein.

If FUBP1 acts as a binding protein for N_m_, then its binding should be affected by the depletion of N_m_. To test this, we conducted *FBL* KD. As PAR-CLIP provides mutation-based identification with less reliable measurement of differential peaks, we performed cross-linking immunoprecipitation sequencing (CLIP-seq) of FUBP1 following *FBL* depletion. Consistent with our PAR-CLIP results, FUBP1 CLIP-seq peaks were predominantly located within intronic regions and protein-coding genes (fig. S5, B and C). Differential peak analysis revealed a decrease in FUBP1 peak signals overlapping with N_m_ sites, while the overall peak intensity remained largely unchanged (Fig. 5C). Similarly, gene-based differential binding measured by FUBP1 RNA immunoprecipitation sequencing (RIP-seq) also demonstrated a significant decrease in N_m_-modified genes compared to unmodified ones after *FBL* KD (Fig. 5D). These findings collectively confirmed that FUBP1 binding to N_m_-modified RNA is affected by the presence of N_m_ modification.

With FUBP1 CLIP-seq identifying valid N_m_-binding sites, we further designed N_m_-modified oligos based on FUBP1 target sequences responsive to N_m_ depletion. EMSA revealed enhanced binding of FUBP1 to N_m_-modified oligos compared to their controls (Fig. 5E and fig. S5D), consolidating FUBP1 as an N_m_-binding protein. Notably, both oligos harboring multiple N_m_ sites showed more pronounced differences in binding affinity compared to single-modified probes (Fig. 3A), suggesting that densely clustered N_m_ may facilitate the recruitment of N_m_-binding proteins.

After confirming FUBP1 as a caRNA N_m_-binding protein, we aimed to explain the N_m_-dependent splicing regulation by FUBP1. FUBP1 facilitates bridging of 5′ and 3′ splice sites (*32*), and its depletion primarily induces exon skipping (*29*). Consistent with this, we knocked down *FUBP1* in HepG2 cells (fig. S5E) and observed that SE was the main AS event (Fig. 5F). This aligns with the N_m_-dependent splicing regulation observed after *FBL* and *U3* KD (Fig. 4H and fig. S4G). Among the SE events, up-regulation occurred more frequently than down-regulation (fig. S5F), consistent with the biased up-regulation induced by *FBL* or *U3* depletion (fig. S4H). To further elucidate the relationship between N_m_ modification, FUBP1 binding, and splicing changes, we examined SE events in genes with caRNA N_m_ modifications. We found that N_m_-modified genes showed greater up-regulation of SE events after FUBP1 depletion than nonmodified genes, supporting that FUBP1 mediates N_m_-dependent inhibition of exon skipping. (fig. S5, G and H). Similarly, genes with reduced FUBP1 binding following *FBL* depletion exhibited higher SE up-regulation after *FUBP1* KD (Fig. 5G), further reinforcing the causal connection between N_m_ modification, FUBP1 binding, and splicing changes.

## DISCUSSION

N_m_ modifications affect the physical properties of mRNA, exerting crucial regulation on the translation efficiency of modified genes (*18*). However, the “reader” proteins of N_m_ modifications on protein-coding genes remain elusive. Characterization of N_m_-binding proteins could reveal N_m_-dependent function. In this study, we used RNA affinity purification, followed by LC-MS/MS to identify N_m_-binding proteins. Immunoblotting validated the enrichment of FUBP1, FUBP3, IGF2BP1, IGF2BP3, FMRP, and NCOA5. This is consistent with previous reports that IGF2BP1, IGF2BP3, and FMRP showed colocalized RNA binding with N_m_ modifications (*28*). Published eCLIP datasets of FUBP3, IGF2BP1, and IGF2BP3 further supported their enrichment around endogenous mRNA N_m_ sites. While functions of IGF2BP proteins align with the effect of mRNA N_m_ on RNA expression levels, the identified FUBP family proteins and NCOA5 suggest unrecognized realms of nuclear N_m_ regulation. However, without quantitative biochemical validation, the enrichment of these proteins could be indirect, and their roles as N_m_-binding proteins need further validations.

We focused on FUBP1 and validated its role as an N_m_-binding protein. EMSA confirmed the direct interaction between FUBP1 and N_m_-modified RNA, while PAR-CLIP confirmed enrichment of FUBP1 around internal mRNA N_m_ sites. To further examine nuclear functions of N_m_ mediated through FUBP1, we profiled N_m_ modification sites on caRNA by N_m_-mut-seq. We identified 5575 caRNA N_m_ sites, with more than half of caRNA N_m_ sites in protein-coding genes localized in introns. We found that intronic N_m_ displayed enrichment around 5′ and 3′ splice sites, suggesting N_m_-mediated splicing regulation. Previous snoKARR-seq data revealed that *U3* is the predominant snoRNA responsible for N_m_ installation on caRNA. Depletion of *U3*, as well as methyltransferase *FBL*, led to up-regulation of SE events, supporting a role of N_m_ in splicing regulation. Prior studies have shown that ectopic installation of N_m_ at splicing branch sites by snoRNA can alter the splicing of certain transcripts (*43*, *44*). Our findings support the N_m_-mediated splicing regulation, pointing to the potential of snoRNA mimics as tools for targeted splicing modulation.

With the profiled N_m_ sites on caRNA, we validated the overlap of FUBP1 binding with caRNA N_m_ sites. To further explore the causal relationship, we disrupted N_m_ by *FBL* depletion and detected impaired FUBP1 binding at N_m_-modified regions by CLIP-seq and RIP-seq. This further supports FUBP1 as the caRNA N_m_-binding protein. *FUBP1* depletion up-regulates SE events preferentially in N_m_-modified genes and genes with down-regulated FUBP1 binding after *FBL* depletion, confirming its role in mediated N_m_-dependent splicing regulation. Overall, our findings identify FUBP1 as a caRNA N_m_-binding protein and uncover a new function of N_m_ in splicing modulation through FUBP1. Nevertheless, it is likely that FUBP1 also contributes to other regulatory processes upon N_m_ binding, given that it was initially characterized as a transcriptional regulator.

A total of 2228 identified N_m_ sites were densely populated on 323 protein-coding genes, suggesting cooperation among N_m_ sites in recruiting binding proteins. This may compensate for the modest preference of FUBP1 for a single N_m_ modification observed in EMSA experiments, contributing to notable enrichment of FUBP1 at N_m_-modified regions. EMSA using densely modified oligos demonstrated a stronger preference of FUBP1 for N_m_ modifications. These observations highlight the importance of quantitative analysis in studies of N_m_-mediated protein recruitment, as a single N_m_ may exert weaker effect compared to densely clustered N_m_ sites. Further investigation is needed to elucidate the underlying mechanism of the weak but additive role of N_m_ in recruiting its potential binding proteins.

### Limitations of the study

The N_m_-binding protein candidates we identified, aside from FUBP1, were not fully validated. More quantitative biochemical evidence supporting their interactions with N_m_ are required in the future. Moreover, the binding affinity of FUBP1 to individual N_m_ sites appears relatively weak compared to that of canonical “readers” of RNA modifications such as m^6^A. Therefore, additional caution and context are needed when interpreting FUBP1 as an N_m_-binding protein.

## MATERIALS AND METHODS

### Cell culture

HepG2 cells (American Type Culture Collection, HB-8065) were cultured with medium containing Dulbecco’s modified Eagle’s medium (Gibco, 11995040), 10% fetal bovine serum (Gibco, 2614079), and 1% penicillin-streptomycin (Gibco, 15140122) at 37°C with 5% CO_2_ in the environment. Cells were passaged when reaching ~90% confluency at 1:4 ratio. Mycoplasma were tested by PCR with primers gggagcaaacaggattagataccct and tgcaccatctgtcactctgttaacctc every half a year.

In the small interfering RNA (siRNA)–mediated KD assay, cells at ~90% confluency were passaged at 1:4 ratio into 15-cm plates. Within 16 hours after passaging, 60 μl of Lipofectamine RNAiMAX Transfection Reagent (Invitrogen, 13778150) and 200 pmol of siRNA were diluted in 1 ml of Opti-MEM I Reduced Serum Medium (Gibco, 31985070), respectively. The solutions were mixed together and incubated at room temperature for 5 min before being added into the cell culture. KD reactions for fewer cells were scaled down on the basis of the bottom area of culture plates. The siRNA used in this paper include siControl (QIAGEN, 1027310), si*FBL* (QIAGEN, SI04164951), siControl2 (Invitrogen, 4390846), and si*FUBP1* (Invitrogen, s16966). Cells were harvested 48 hours posttransfection. KD efficiency was measured by qPCR with primers from Origene (HP205317).

*U3* KD was conducted with *U3* allele-specific oligonucleotide (ASO) (mU*mC*mA*mC*mC*T*T*C*A*C*C*C*T*C*T*mC*mC*mA*mC*mU) controlled by green fluorescent protein ASO (mC*mU*mG*mC*mC*A*T*C*C*A*G*A*T*C*G*mU*mU*mA*mU*mC). Transfection was done by Lipofectamine 3000 transfection reagents (Invitrogen, L3000015), where 600 pmol of ASO and 8 μl of P3000 reagents were added to 125 μl of Opti-MEM medium. In parallel, 6 μl of Lipofectamine 3000 reagents were diluted in 125 μl of Opti-MEM medium. The two mixtures were mixed together, incubated at room temperature for 10 min, and then added to 2 ml of medium in a six-well plate. HepG2 cells corresponding to one-third of a 10-cm plate at ~90% confluency were then added to the transfected medium. Cells were cultured for 72 hours before harvest. KD efficiency was measured by qPCR primer pair CGTGTAGAGCACCGAAAACC and CACTCAGACCGCGTTCTCTC.

### Immunoblotting

Samples were lysed in 2× NuPAGE LDS sample buffer (Invitrogen, NP0007) supplemented with 1:20 (v/v) 2-mercaptoethanol (Sigma-Aldrich, M6250-1L). After incubation at 95°C for 10 min, the denatured samples were loaded into 4 to 12% NuPAGE bis-tris gels (Invitrogen, NP0322BOX) and transferred onto nitrocellulose membranes (Bio-Rad, 1620115). Samples were blocked by 5% bovine serum albumin (BSA) (Fisher Scientific, BP1600-1) in PBS Tween-20 (PBST; Thermo Fisher Scientific, 28352) for 30 min, followed by overnight incubation at 4°C in primary antibodies with designated dilution ratios in 3% BSA diluted by PBST. Membranes were washed three times and incubated in the secondary antibody conjugated to HRP at room temperature for 1 hour. Protein signals were developed by SuperSignal West Dura Extended Duration Substrate (Thermo Fisher Scientific, 34075). Antibodies used in this study and their dilution ratios are as follows: anti-FUBP1 (Abcam, ab192867; 1:1000), anti-FUBP3 (Abcam, ab181025; 1:1000), anti-IGF2BP1 (MBL International, RN007P; 1:1000), anti-IGF2BP3 (MBL International, RN009P; 1:1000), anti-NCOA5 (Proteintech, 20175-1-AP; 1:500), anti-FMRP (Abcam, ab17722; 1:1000), and anti-rabbit immunoglobulin G (IgG) linked with HRP (Cell Signaling Technology, 7074S; 1:2000).

### RNA affinity purification

The experiment followed the protocol in the previous publication (*45*) with adjustment. A total of 30 μl of Dynabeads MyOne Streptavidin C1 bead suspension was washed with RNA binding buffer [50 mM Hepes (pH 7.5), 150 mM NaCl, 0.5% NP-40 substitute, and 10 mM MgCl_2_] and incubated in RNA binding buffer supplemented with yeast tRNA (100 μg/ml; Invitrogen, AM7119) for 1 hour at 4°C with rotation. After two rounds of washing, 400 pmol of N_m_-modified or control probes were incubated with beads suspended in RNA binding buffer for 30 min at 4°C with rotation. Beads conjugated with oligos were washed with RNA wash buffer [50 mM Hepes (pH 7.5), 250 mM NaCl, 0.5% NP-40 substitute, and 10 mM MgCl_2_] and then with protein incubation buffer [10 mM tris-HCl (pH 7.5), 150 mM KCl, 1.5 mM MgCl_2_, 0.1% NP-40 substitute, and 0.5 mM dithiothreitol (DTT)] twice. HepG2 cell pellets in the volume of 45 μl were lysed in 400 μl of lysis buffer [50 mM tris (pH 7.5), 100 mM NaCl, 1% NP-40 substitute, 0.5% sodium deoxycholate, 100× proteinase inhibitor cocktail (Sigma-Aldrich, P8340), and 100× SUPERase·In RNase (ribonuclease) Inhibitor (Invitrogen, AM2696)] for 30 min at 4°C with rotation. The supernatant of the lysate was harvested by centrifugation at 12,000*g* for 15 min and separated to two equal halves after saving 5% as input. The lysate was incubated with beads conjugated with oligos, supplemented with tRNA (50 μg/ml), 0.5 mM DTT, and 100× SUPERase·In, and incubated at room temperature for 30 min and 4°C for 90 min with rotation. The beads were washed three times with protein incubation buffer before removal of all supernatant.

### LC-MS/MS analysis

Samples were prepared with three replicates, harvested on dry beads, and frozen on dry ice when shipped to the MS center. The beads were heated in 3× reducing LDS sample buffer with 15 mM DTT and 2 M biotin for 10 min at 95°C, and the supernatant was loaded on 12% bis-tris propane SDS–polyacrylamide gel electrophoresis gel for removal of detergent. The gel was run shortly and stained with colloidal Coomassie blue for gel cut of the whole lane. Gel pieces were reduced with DTT, alkylated with iodoacetamide, washed properly, and digested with trypsin overnight at 37°C. The extracted peptides were dried down, redissolved in 2.5% acetonitrile-water solution with 0.1% formic acid, and then run by nano–LC-MS/MS using a 2-hour gradient on a 0.075 mm–by–250 mm C18 column feeding into an Orbitrap Eclipse mass spectrometer.

The quantitation was done by Proteome Discoverer (Thermo Fisher Scientific; version 2.4). All MS/MS samples were searched with Mascot (Matrix Science, London, UK; version 2.6.2) using cRAP_20150130.fasta (124 entries) and uniprot-human_20201207 database (75777 entries) assuming trypsin digestion, with a fragment ion mass tolerance of 0.02 Da and a parent ion tolerance of 10.0 parts per million. The specified variable modifications included asparagine and glutamine deamidation, methionine oxidation, lysine and arginine methylation, and cysteine carbamidomethylation. Peptides were validated by Percolator with a 0.01 posterior error probability threshold. The data were searched with a decoy database to set the false discovery rate (FDR) to 1%. The peptides were quantified using the precursor abundance based on intensity, with the peak abundance normalized by total peptide amount. The sum of peptide group abundances for each sample were normalized to the maximum sum of the analyzed samples. The protein ratios were calculated using summed abundance for each replicate separately and the geometric median of the resulting ratios was used as the protein ratios. To compensate for missing values in some of the replicates, the replicate-based resampling imputation mode was used. The significance of differential expression was generated by analysis of variance (ANOVA) test, and the *P* values were adjusted by the Benjamini-Hochberg method.

### Protein purification

FUBP1-strep were expressed in 100 ml of Expi293F cells (Gibco, A14527) for 48 hours, lysed in lysis buffer [20 mM tris (pH 8.0), 150 mM KCl, 2 mM EDTA, 100× phenylmethylsulfonyl fluoride (PMSF), 1% NP-40 substitute, and 0.5 mM DTT] at 4°C for 30 min. Supernatant was harvested by centrifugation and diluted to threefold volume before passing through 0.22-μm filter. Samples were loaded to 200 μl of Strep-Tactin Sepharose resin (IBA, 21201010) and incubated at 4°C for 1 hour. The resin was flowed through by lysate twice and washed with 25 ml of wash buffer [50 mM tris (pH 8.0), 250 mM NaCl, 0.05% NP-40 substitute, 0.5 mM DTT, and 100× PMSF]. Protein was eluted six times with elution buffer [50 mM tris, (pH 8.0), 250 mM NaCl, 0.01% NP-40 substitute, 0.5 mM DTT, and 2.5 mM desthiobiotin] with 100 μl each time, concentrated with 30-kDa Amicon Ultra Centrifugal Filter (Millipore, UFC503008), and exchanged to storage buffer [50 mM tris, (pH 8.0), 150 mM KCl, 0.1 mM EDTA, and 20% glycerol]. The purified protein was aliquoted, snap frozen in liquid nitrogen, and stored at −80°C.

### Electrophoretic mobility shift assay

Probes for EMSA were labeled with 6-carboxyfluorescein (FAM) fluorophore at the 5′ end, with sequence noted in the figures. Probes were refolded by incubation at 75°C for 2 min and natural cooling to room temperature for 10 min. FUBP1 proteins of designated concentrations were incubated with FAM-labeled oligos in binding buffer [10 mM tris (pH 7.5), 140 mM KCl, 10 mM NaCl, 1 mM MgCl_2_, 10% glycerol, 1 mM DTT, and SUPERase·In RNase Inhibitor (1 U/μl)] at room temperature for 30 min. The mixtures were loaded to 4 to 20% Novex tris-borate EDTA (TBE) gels (EC62255BOX) that have been rerun at 90 V for 30 min at 4°C in 0.5× TBE. Gels were run for 2 hours before imaging. The fluorescence signal intensity was quantified by ImageJ, and *K*_d_ value was calculated by GraphPad.

### UHPLC-QQQ-MS/MS quantification

HepG2 mRNA were purified by two rounds of polyadenylate [poly(A)] selection following the commercial protocol of Dynabeads mRNA DIRECT Purification Kit (Invitrogen, 61011). HepG2 chromatin were purified following the published protocol reported previously (*7*), with adjustment of lysis buffer to 10 mM tris-HCl (pH 7.5), 0.15% NP-40 substitute, and 150 mM NaCl. The caRNA were purified from fractionated chromatin, followed by two rounds of RiboMinus reactions according to the commercial instruction of RiboMinus Eukaryote System v2 (Invitrogen, A15026).

The verification of rRNA contamination was performed by qPCR for 18*S* (with primer pair CGGACAGGATTGACAGATTG and CAAATCGCTCCACCAACTAA) and 45*S* (with primer pair TCGCTGCGATCTATTGAAAG and AGGAAGACGAACGGAAGGAC) rRNA. The same amount of RNA was used for RT. The absolute amount of cDNA product was quantitated by the control of PCR product of the two primer pairs with known amount of DNA template.

For UHPLC-QQQ-MS/MS detection, 100 ng of RNA was digested with Nuclease P1 [New England Biolabs (NEB), M0660S] at 37°C overnight, followed by digestion with shrimp alkaline phosphatase (rSAP, NEB, M0371S) in rCutSmart buffer 2 hours at 37°C. The samples were diluted and filtered by 0.22-μm polyvinylidene difluoride filter (Millipore, SLGVR33RB) and then injected into a C18 reversed phase column coupled online to Agilent 6460 LC-MS/MS spectrometer in positive electrospray ionization mode. Nucleosides were quantified using nucleoside-to-base transitions of A_m_ (282 to 136), A (268 to 136), G_m_ (198.1 to 152.1), G (284 to 152), C_m_ (258.2 to 112), and C (244 to 112). The signal intensity of N_m_ nucleotides was normalized to the corresponding unmodified nucleotides to enable comparison of samples with different length.

### Photoactivatable ribonucleoside-enhanced cross-linking and immunoprecipitation

The experiment was performed with an adapted protocol from a previous report (*46*). Two biological replicates of 150 million HepG2 cells were cultured with 200 μM 4-Thiouridine for 14 hours. Cells were cross-linked by 365-nm ultraviolet (UV) at 1500 J/m^2^ twice, harvested, and lysed by iCLIP lysis buffer [50 mM tris (pH 7.5), 100 mM NaCl, 1% NP-40 substitute, 0.1% SDS, 0.5% sodium deoxycholate, 100× protease inhibitor cocktail, and 100× SUPERase·In RNase Inhibitor] at 4°C for 15 min with rotation. To release FUBP1 proteins associated with the chromatin, cell lysate was supplemented with 1% SDS, sonicated at 30% amplitude with 2-s:4-s cycles for 1 min on ice. The lysate was 10-fold diluted by iCLIP lysis buffer without SDS, centrifuged to save the supernatant, and treated with RNase T1 (0.2 U/μl; Thermo Fisher Scientific, EN0642) at 22°C for 15 min before being quenched on ice for 5 min. Protein G beads (Invitrogen, 10009D) were conjugated with 20 μg of FUBP1 antibody (Abcam, ab192867) or rabbit IgG (Cell Signaling Technology, 2729) by incubation at 4°C for 1 hour, then washed, and mixed with RNase T1–treated lysate to rotate at 4°C for 4 hours. Beads were washed three times with CLIP wash buffer [50 mM tris (pH 7.5), 300 mM KCl, 0.05% NP-40 substitute, 1000× proteinase inhibitor cocktail, and 1000× SUPERase·In RNase Inhibitor], digested with RNase T1 (10 U/μl) at 22°C for 8 min, washed with CLIP high-salt buffer [50 mM tris (pH 7.5), 500 mM KCl, 0.05% NP-40 substitute, 1000× protease inhibitor cocktail, and 1000× SUPERase·In RNase Inhibitor] and T4 polynucleotide kinase (T4 PNK) buffer without DTT three times each, and underwent end repair by T4 PNK (NEB, M0201L). The immunoprecipitation was validated by biotinylation, eluted with proteinase K digestion (Thermo Fisher Scientific, EO0491), with purified RNA used to construct libraries with NEBNext Small RNA Library Prep Set (NEB, E7330S). Sequencing was performed by Illumina NovaSeq 6000 paired-end 50-bp reads.

### Bioinformatic analysis of PAR-CLIP

For both FUBP1 PAR-CLIP and its IgG control, adapters were trimmed by Cutadapt (*47*), and reads were mapped to hg38 human genome by HISAT2 (*48*), with parameter “--reorder --no-unal --pen-noncansplice 12.” Duplicates were filtered by Picard MarkDuplicates. R1 reads with mapping quality higher than 30 were used for identification of FUBP1-binding clusters by wavClusteR (*49*, *50*), with removal of reads containing “^” in the MD tag. The identified clusters were overlapped and then annotated by Homer (*51*) annotatepeaks. The binding clusters identified by IgG were supposed to be removed from the FUBP1-binding clusters. Nevertheless, we identified no clusters from IgG control. Metaplots centering at N_m_ sites were generated by deepTools (*52*). Motifs were identified by Homer findMotifsGenome with parameter “-rna -size 200 -len 6”.

### Cross-linking immunoprecipitation sequencing

The experiment followed the procedure in the previous publication (*53*). Three biological replicates of 150 million HepG2 cells were harvested after 48 hours of FBL KD, with the KD efficiency validated by RT-qPCR with primers from Origene (HP205317). Samples were cross-linked three times by UV (1500 J/m^2^) at 254 nm, lysed, centrifuged, and digested with the same condition as PAR-CLIP. After saving 2% of the lysate as input, lysates were incubated with beads treated the same as PAR-CLIP. The following steps were exactly the same, with the exception that input samples were treated separately with RNase T1 (10 U/μl) at 22°C for 8 min, followed by proteinase K digestion and end repair in the next. Sequencing by Illumina NovaSeq X was conducted for single-end 100-bp reads.

### Bioinformatic analysis of CLIP-seq

Cutadapt (*47*) was used to trim the adapters, and HISAT2 (*48*) mapping to the reference genome (hg38) was performed, with parameter “--reorder --no-unal --pen-noncansplice 12.” Peaks were identified by Piranha (*54*) with input samples as the covariate, and bin size designated as 50 bp. The identified peaks of siControl and siFBL were merged and annotated by Homer (*51*) annotatepeaks. Differential binding was analyzed by DiffBind (*55*), with relative log expression normalization considering the background (normalize = DBA_NORM_RLE, background = TRUE). Down-regulated peaks were defined as a log_2_ fold change of <−0.58 and an FDR < 0.1. Peaks within 2 kb of N_m_ sites were considered as N_m_ modified peaks for computing binding fold change.

### RNA sequencing

HepG2 cell RNA was harvested by TRIzol reagent (Invitrogen, 15596026) after 48 hours of siRNA-mediated KD or 72 hours of ASO-mediated KD with three biological replicates. RNA was purified with the manufacturer’s procedures, and the KD was measured by RT-qPCR with primers from Origene (HP207343). After poly(A) RNA selection with Dynabeads mRNA DIRECT Purification Kit and the samples were constructed to libraries with SMARTer Stranded Total RNA-Seq Kit v2–Pico Input Mammalian (Takara, 634412). Next-generation sequencing was performed with Illumina NovaSeq 6000 paired-end 150-bp reads.

### RNA immunoprecipitation sequencing

Two biological replicates of HepG2 cells were harvested after 48 hours of *FBL* KD. The KD efficiency was validated by RT-qPCR. Cells were lysed by iCLIP lysis buffer at 4°C for 15 min with rotation, followed by centrifugation at 13,000*g* for 15 min. Protein G beads were conjugated with 16 μg of FUBP1 antibody by incubation at 4°C for 1 hour, then washed, and mixed with lysate supernatant to rotate at 4°C for 4 hours. Beads were washed three times with CLIP wash buffer and eluted with proteinase K digestion (Thermo Fisher Scientific, EO0491) Input samples harvested before immunoprecipitation was also digested with proteinase K for RNA recovery. The purified RNA was constructed for library with SMARTer Stranded Total RNA-Seq Kit v2–Pico Input Mammalian. Next-generation sequencing was performed with Illumina NovaSeq 6000 paired-end 150-bp reads.

### Bioinformatic analysis for RNA sequencing and RIP-seq

Adapters were trimmed by Cutadapt (*47*), and reads were mapped to hg38 human genome by HISAT2 (*48*), with parameter “--reorder --no-unal --pen-noncansplice 12.” Reads were counted for each gene by HTSeq (*56*), and differential expression analysis by DESeq2 (*57*) was conducted. For RIP-seq samples, the differential expression analysis compares immunoprecipitation versus input samples, where the fold change was considered as enrichment of each gene. The enrichment was further compared between control and FBL KD samples to measure the differential binding of FUBP1 upon N_m_ depletion.

The AS events were detected by rMATS (*58*), where we used the input samples of RIP-seq for analysis upon *FBL* KD. Only junction reads were used for calculation of AS scores. Events with an FDR of <0.1 were considered as events.

### Nm-mut-seq

Three biological replicates of HepG2 caRNA were fractionated and purified in the same procedures as UHPLC-QQQ-MS/MS analysis. rRNA was removed by RiboMinus Eukaryote System v2, and the residual RNA was cleaned up by RNA Clean & Concentrator-5 (Zymo Research, R1014) with removal of a short RNA of <200 nt. The construction of Nm-mut-seq followed the published procedures (*27*), using 3′ linker 5’rApp-NNNNNATCACGAGATCGGAAGAGCACACGTCT-3SpC3 and 5′ SR adapter supplied in the NEBNext Small RNA Library Prep Set. Sequencing by Illumina NovaSeq X was conducted with single-end 100-bp reads.

### Bioinformatic analysis for Nm-mut-seq

Adapters were trimmed by Cutadapt (*47*), and duplicates marked by unique molecular identifier (UMI) was removed using BBMap (*59*). Reads were mapped to hg38 human genome by TopHat2 (*60*), with parameters “-g 4 -N 3 --read-edit-dist 3.” Mutations to T were identified by JACUSA (*39*), with further selection of mutated sites in all three replicates having a sequencing depth of ≥10, a mutated read depth of ≥3, mutation ratios of >3-fold of input mutation ratios, and mutation ratios of >1.5-fold of background mutation ratios. N_m_ sites were annotated by Homer (*51*) annotatepeaks.

### Statistical analysis

*P* values annotated in figures were quantified on the basis of two-tailed Student’s *t* test. Chi-squared test was used to statistically test the preferential occurrence of different splicing events with increased or decreased inclusion level differences. The *K*_d_ value and SD for EMSA were fitted by GraphPad Prism under the mode of “one site–specific binding with Hill slope.”
