# Supplementary material for: Nuclear 2′-O-methylation regulates RNA splicing through its binding protein FUBP1
Source: Sci Adv. 2025 Oct 17;11(42):eady3894. doi: 10.1126/sciadv.ady3894 (PMC12533644; doi:10.1126/sciadv.ady3894)
Supplement: Supplementary file 1 — Figs. S1 to S5 Legends for tables S1 to S10 [file sciadv.ady3894_sm.pdf]

Science Advances
AAAS

Supplementary Materials for

Nuclear 2′-*O*-methylation regulates RNA splicing through its binding protein FUBP1

Boyang Gao *et al.*

Corresponding author: Chuan He, chuanhe@uchicago.edu

Sci. Adv. **11**, eady3894 (2025)
DOI: 10.1126/sciadv.ady3894

The PDF file includes:

Figs. S1 to S5
Legends for tables S1 to S10

Other Supplementary Material for this manuscript includes the following:

Tables S1 to S10

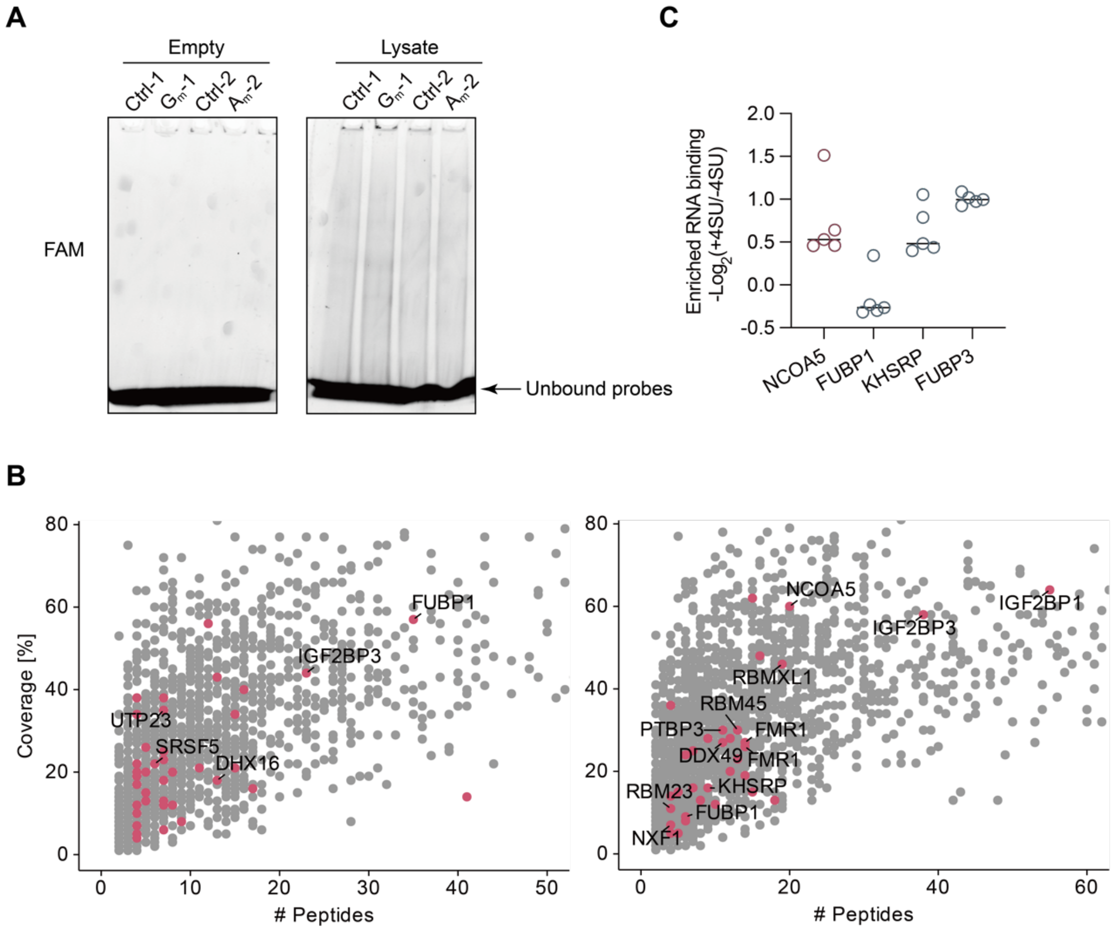

Fig. S1. Identification of tentative N_m-binding proteins.

(**A**) Imaging of fluorescein amidite (FAM)-labeled RNA oligos following UV-crosslinking with cell lysate (Lysate) or water (Empty). (**B**) Proteins that showed enriched binding to RNA G_m-1 (left) and A_m-2 (right) probes. Red: Tentative N_m-binding proteins. # Peptides: peptide number. Coverage: peptide coverage of the protein. (**C**) Enriched RNA binding of 5 most highly enriched peptides bound to RNA represented by -$\log_2$ fold change of +4SU/-4SU samples in published RBR-ID of K562 cells. Peptides were filtered for adjusted p-value < 0.05 and ranked with fold change.

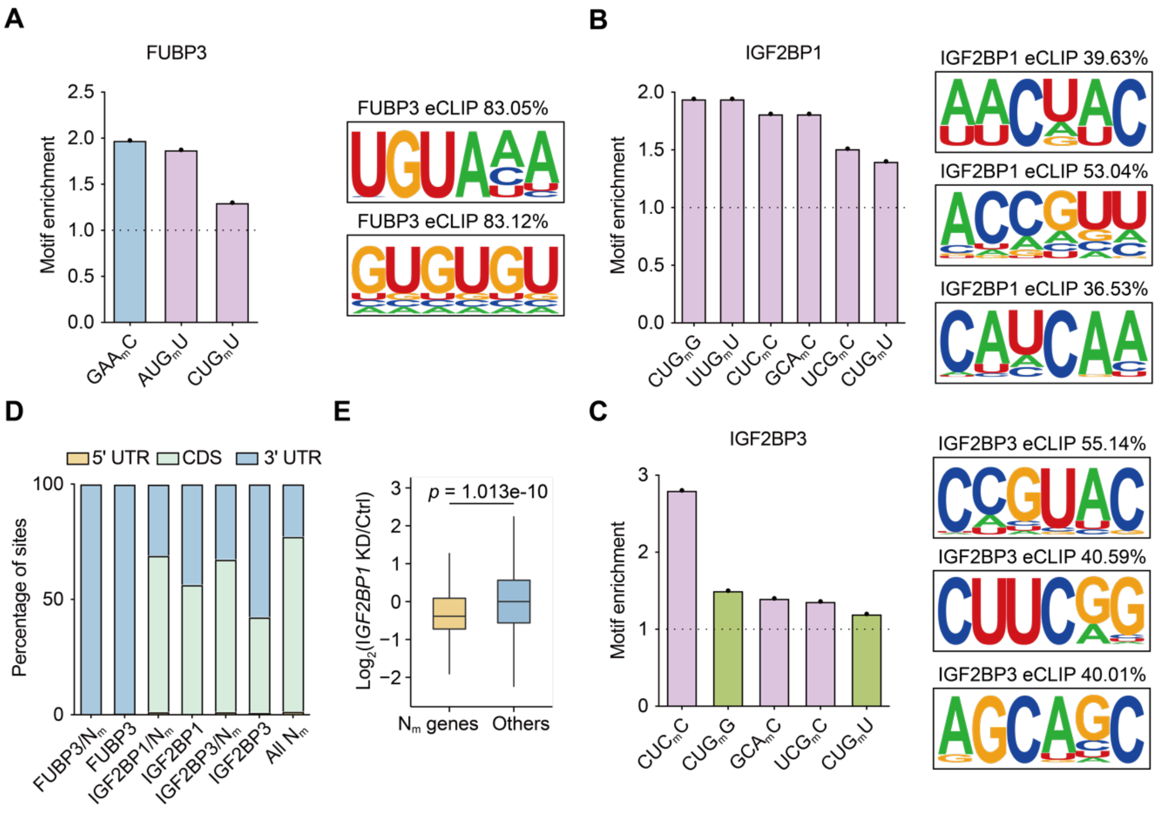

Fig. S2. The target properties of N$_m$-binding protein candidates.

(**A-C**) Enrichment of N$_m$ motifs at the N$_m$-modified binding sites (left) and the enriched motifs across all binding sites from ENCODE eCLIP dataset (right) of FUBP3 (A), IGF2BP1 (B), and IGF2BP3 (C). N$_m$ motifs resembling the enriched RNA binding motifs of the respective RBPs are shown in pink. N$_m$ motifs that are also enriched by other members of the protein families (IGF2BP1/IGF2BP3) are colored in green. (**D**) Distribution of N$_m$ sites bound by each N$_m$-binding protein candidate (protein name/N$_m$), total binding peaks of each N$_m$-binding protein candidate, or all N$_m$ sites across different mRNA regions. (**E**) Fold changes in RNA expression following *IGF2BP1* KD for genes with confident N$_m$ modification (N$_m$ genes) versus other genes (Others).

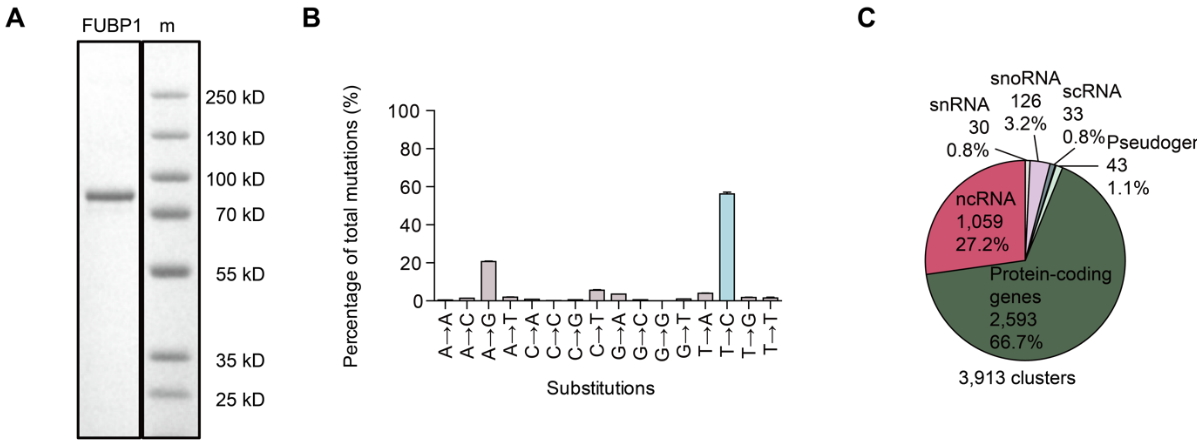

Fig. S3. Analysis of FUBP1 binding profile.

(**A**) Coomassie staining of purified FUBP1-strep expressed in Expi293F cells. (**B**) Base conversion ratios in FUBP1 PAR-CLIP. (**C**) Distribution of FUBP1 PAR-CLIP clusters across different gene types.

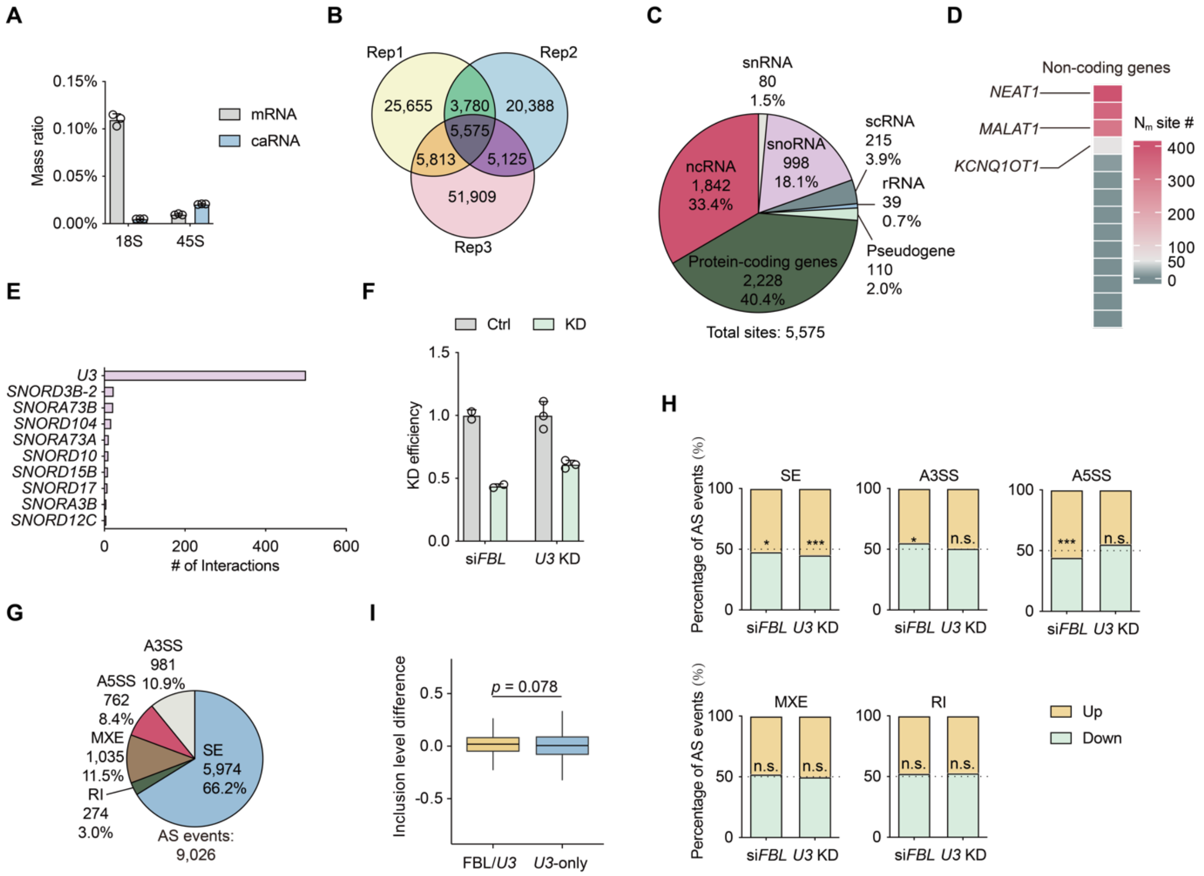

Fig. S4. caRNA N_m distribution and its implied function in splicing regulation.

(**A**) Mass ratios of 18S and 45S rRNA contamination in mRNA and caRNA after two rounds of ribodepletion, quantified by qPCR. (**B**) Overlap of identified caRNA N_m sites from 3 replicates. (**C**) Distribution of 5,575 caRNA N_m sites across different gene types. (**D**) Heatmap showing numbers of N_m sites in various non-coding caRNAs. (**E**) Number of snoRNA-caRNA interactions detected by snoKARR-seq at N_m sites within protein-coding genes. (**F**) KD efficiency measured by qPCR normalized to *ACTB*. (**G**) Profile of *U3*-dependent AS events identified with FDR < 0.1. (**H**) Percentage of increased (Up) or decreased (Down) AS events after FBL depletion or *U3* KD. *, *p*-value < 0.05; ***, *p*-value < 0.005; n.s., not significant. (**I**) Inclusion level differences of FBL/*U3* or *U3*-only SE events.

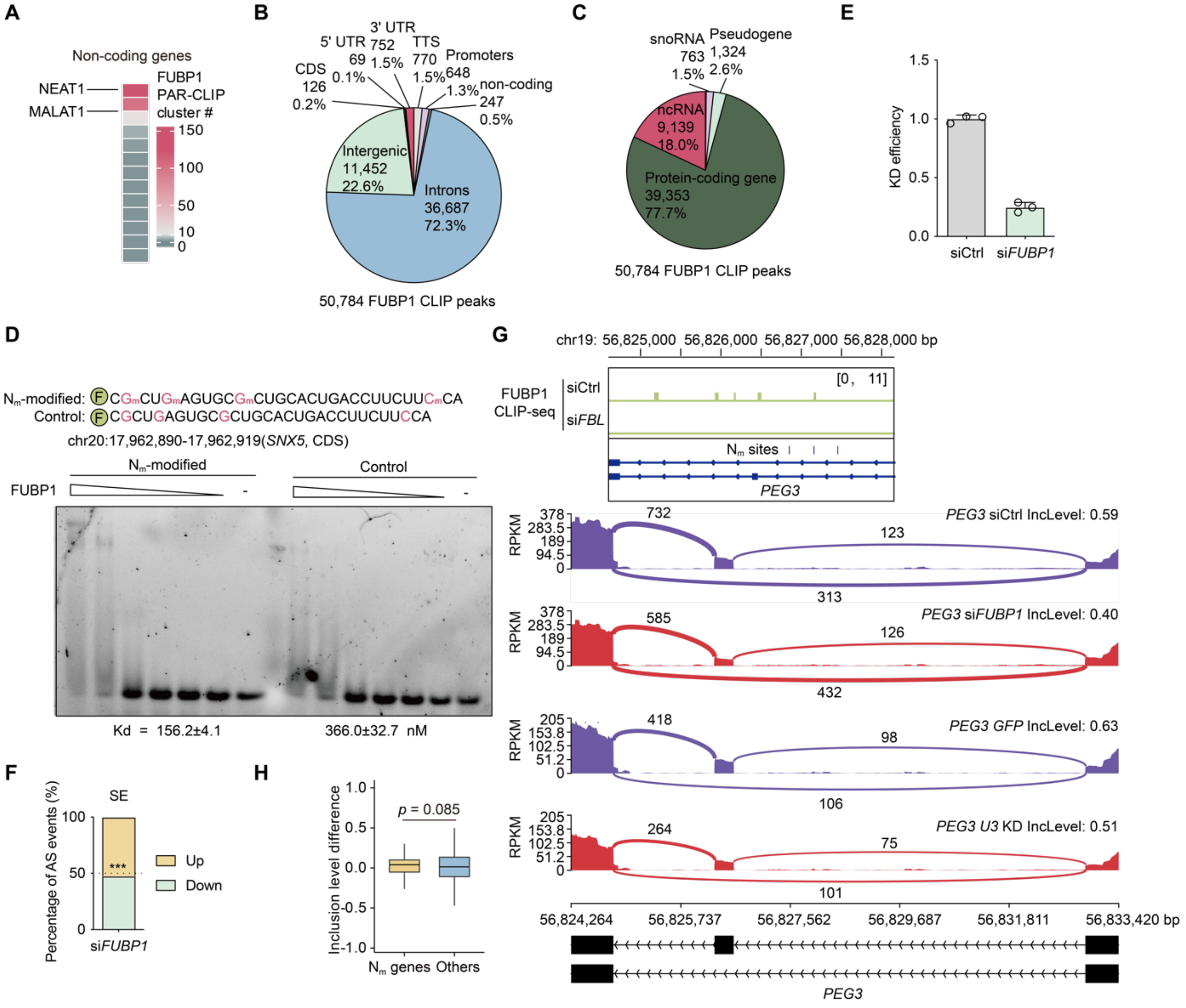

Fig. S5. FUBP1 binds to N_m-modified caRNA to affect splicing.

(**A**) Heatmap showing numbers of FUBP1 PAR-CLIP clusters in various non-coding RNA. (**B**) Distribution of FUBP1 CLIP-seq peaks across transcript elements. (**C**) Distribution of FUBP1 CLIP-seq peaks across gene types. (**D**) EMSA of FUBP1 binding towards N_m-modified or control probes originated from *SNX5* CDS. Probe concentration: 10 nM. Protein concentration: starting from 400 nM with 2-fold dilution. (**E**) KD efficiency measured by qPCR normalized to *ACTB*. (**F**) Percentage of increased (Up) or decreased (Down) SE events after FUBP1 depletion. ***, p-value < 0.005. (**G**) Representative IGV tracks of differential FUBP1 CLIP-seq after si*FBL* and differential SE following si*FUBP1* or *U3* KD. (**H**) Inclusion level differences of SE events in genes with caRNA N_m sites (N_m genes) versus other genes (Others) after FUBP1 depletion.

Supplementary Tables S1-10

Table S1. Protein enrichment from G_m-1/Ctrl-1 oligo pull down followed by proteomics.
Table S2. Protein enrichment from A_m-2/Ctrl-2 oligo pull down followed by proteomics.
Table S3. Annotation of FUBP1 PAR-CLIP clusters.
Table S4. caRNA N_m sites identified in each replicate by N_m-mut-seq.
Table S5. Gene annotation of caRNA N_m sites identified by N_m-mut-seq.
Table S6. Skipped exons of FBL-depleted HepG2 cells.
Table S7. Skipped exons of *U3*-depleted HepG2 cells.
Table S8. DiffBind analysis of FUBP1 CLIP-seq peaks with FBL depletion.
Table S9. DESeq2 analysis of FUBP1 RIP-seq with FBL depletion.
Table S10. Skipped exons of FUBP1-depleted HepG2 cells.
